# Multifactorial contributors to knee osteoarthritis identified by Machine Learning and Bayesian network analysis: A cross-sectional study of the Iwaki Health Promotion Project

**DOI:** 10.1016/j.ocarto.2026.100874

**Published:** 2026-09-07

**Authors:** Ryo Tomita, Eiji Sasaki, Kyota Ishibashi, Ryoto Kura, Hikaru Kristi Ishibashi, Yuka Kimura, Kenji Fujimoto, Yoshinori Tamada, Koichi Murashita, Yasuyuki Ishibashi

**Affiliations:** a Department of Orthopaedic Surgery, Graduate School of Medicine, Hirosaki University, Hirosaki, Japan; b Department of Electronics and Information Technology, Graduate School of Science and Technology, Hirosaki University, Hirosaki, Japan; c Department of Medical Data Intelligence, Research Center for Health-Medical Data Science, Graduate School of Medicine, Hirosaki University, Hirosaki, Japan; d Research Institute of Health Innovation, Hirosaki University, Hirosaki, Japan

**Keywords:** Knee osteoarthritis, Machine learning, Bayesian network analysis

## Abstract

**Objective:**

To explore factors associated with radiographic knee osteoarthritis (KOA) among a broad range of variables using community-based health examination data through machine-learning-based classification and Bayesian network (BN) analysis.

**Methods:**

This cross-sectional study included 703 participants from the 2022 Iwaki Health Promotion Project. Radiographic KOA was defined as Kellgren–Lawrence grade ≥2 in the right knee. From 993 candidate variables encompassing clinical, imaging, laboratory, lifestyle, and dietary data, machine learning with feature selection was used to develop binary classification models for the overall population and, as a subgroup analysis, women. Model performance was evaluated using 10-fold cross-validation. BN analysis with 1000 bootstrap resamplings was subsequently performed to explore conditional dependence structures among the selected variables and KOA.

**Results:**

Fifty variables in the overall population and 54 in women were retained after feature selection. The classification models achieved AUCs of 0.849 and 0.808, respectively, compared with 0.761 for a reference model including age, sex, and BMI. Variables related to knee symptoms, amino acids, fatty acids, sex hormones, and dietary habits were associated with KOA in the BN analyses. Dietary habit–related variables were particularly prominent in the overall population. The frequencies of carbonated beverage and deep-fried chicken consumption were significantly associated with KOA in logistic regression analysis.

**Conclusions:**

Machine learning combined with BN analysis identified multidomain factors associated with radiographic KOA from comprehensive community health data. These findings are exploratory and require validation in longitudinal and independent cohorts.

## 1 Introduction

Osteoarthritis (OA) is the most common form of arthritis and a leading cause of disability worldwide, constituting a major public health and socioeconomic problem [1]. The prevalence of radiographic knee osteoarthritis (KOA) has been reported to be 42.6% in men and 62.4% in women over 40 years of age [2]. Multiple risk factors for KOA have been identified. Some are non-modifiable, such as age [3], sex [4] and race [3,5], whereas others are modifiable, including obesity [[3], [4], [5]] and lower limb malalignment [3]. However, potential risk factors for KOA have not been fully elucidated, as comprehensive analyses in large-sample cohort study are required.

In recent years, artificial intelligence (AI), particularly machine and deep learning, has been increasingly applied in biomedical research. These approaches have demonstrated promising performance in diagnosing KOA from radiographs and MRI [6], predicting disease progression [7], and forecasting outcomes after total knee arthroplasty [8]. AI-based studies have identified conventional risk factors as significant contributors to prediction models, underscoring their clinical relevance. Nevertheless, most existing AI-based studies have primarily relied on imaging data and a limited set of clinical variables [9]. Comprehensive analyses incorporating lifestyle behaviors, dietary habits, and laboratory biomarkers, including blood and urine test results, remain scarce. This gap highlights the need for a more holistic approach to understand the risk architecture of knee OA.

Therefore, The aim of the present study was to explore factors associated with radiographic KOA among a broad range of variables using community-based health examination data, through the development of a binary classification model followed by Bayesian network (BN) analysis. We hypothesized that, in addition to established conventional risk factors, potential novel determinants such as dietary habits, metabolites and hormonal status could be identified. Any of these factors may potentially be applied as biomarkers during screening or incorporated into preventive intervention programs. Furthermore, to minimize the influence of confounding factors and to explore variables that may act directly on KOA, we employed BN analysis. BN analysis is a probabilistic graphical modeling approach that evaluates relationships among variables based on conditional probabilities and organizes potential causal structures among multiple events in a graphical framework.

## 2 Methods

This cross-sectional study was conducted among volunteer participants of the Iwaki Health Promotion Project [10], a preventive medicine initiative aimed at extending healthy life expectancy by providing health checkups to the general population of the Iwaki area of Hirosaki City, western Aomori Prefecture, Japan. The KOA study began in 2008, with annual examinations conducted to monitor the natural progression of symptoms. Physical examinations, functional tests, laboratory findings, lifestyle information, and imaging data were collected annually. Participants were recruited from the community through mass media advertisements with the assistance of public health nurses. All participants provided written informed consent. The study was conducted in accordance with the 1964 Declaration of Helsinki and its later amendments and was approved by the Ethics Committee of the Hirosaki University Graduate School of Medicine (approval no. 2024-016H).

### 2.1 Participants

**Fig. 1:**
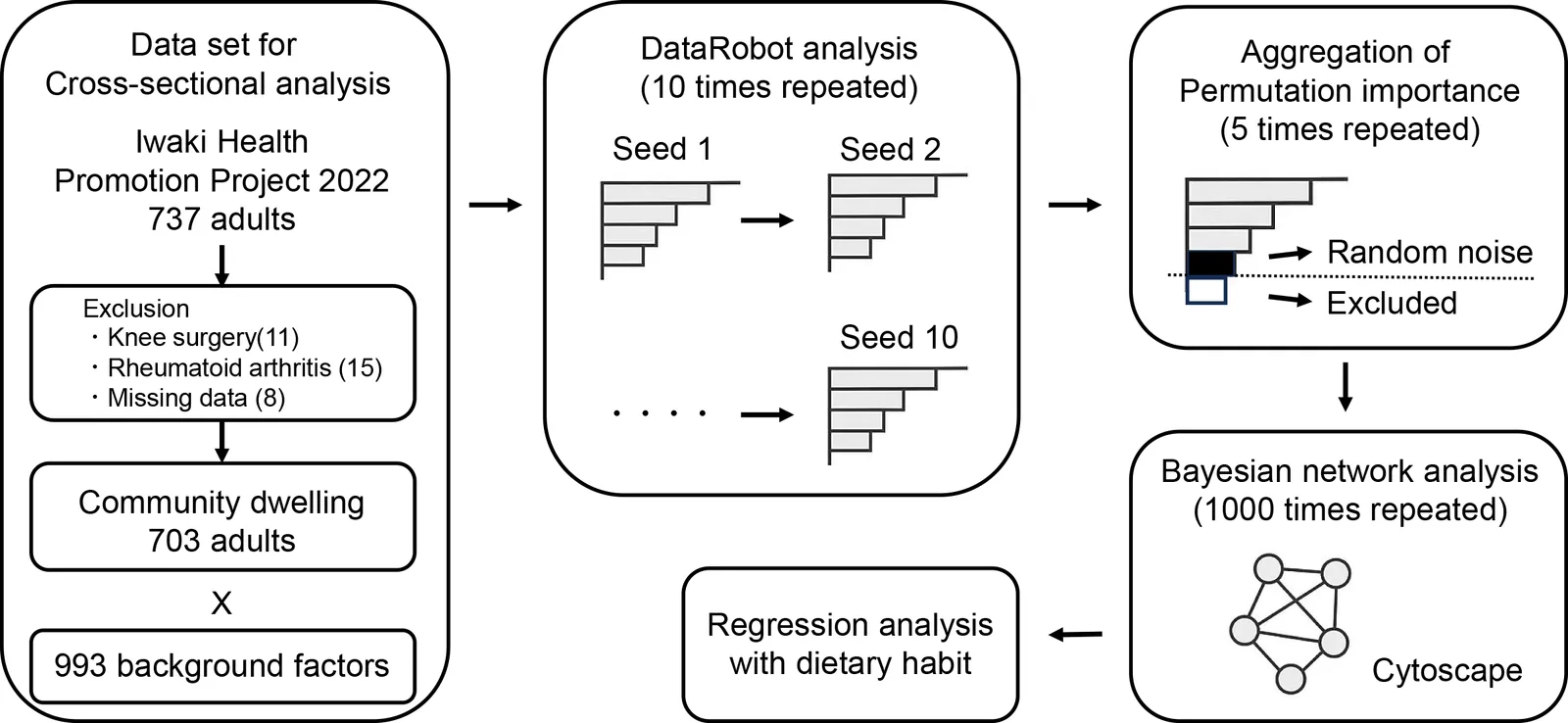
Analysis workflow for robust feature selection and BN construction.

Among approximately 11,000 residents of the Iwaki area, 737 volunteers participated in the Iwaki Health Promotion Project in 2022 and were initially included in this study. Exclusion criteria were: (1) history of knee joint surgery (n = 11), (2) diagnosis of rheumatoid arthritis (n = 15), and (3) missing data (n = 8) (Fig. 1). After exclusions, 703 participants were included in the final statistical analyses.

### 2.2 Radiographic evaluation

Plain-standing radiographs of both knees were obtained using a CXDI-40 E G digital radiography system (Canon Inc., Tokyo, Japan). Full-extension, weight-bearing anteroposterior radiographs with foot map positioning were acquired on the day of examination, along with full-length radiographs of the lower limbs. Imaging parameters were standardized at 60 kV, 50 mA, and 80 ms for all participants. KOA was defined as the presence of radiographic changes corresponding to a Kellgren-Lawrence (KL) grade ≥2 in the right knee. All radiographs were independently graded by two orthopedic surgeons, with an intraclass correlation coefficient (ICC) of 0.821 (*p* < 0.001).

Lower limb alignment was evaluated using mediCAD® software Version 5.5 (TOYO Corporation) [10]. The following radiographic parameters were measured: mechanical lateral distal femoral angle, mechanical medial proximal tibial angle, joint line convergence angle, tibial length, mechanical axis deviation, percentage of mechanical axis (%MA), anatomical mechanical angle, femorotibial angle, femoral bowing angle, and the angle between the femoral shaft axis and the mechanical axis of the tibia (FSAmTA). Using these measurements, joint line obliquity, mechanical hip–knee–ankle angle (mHKA), and arithmetic hip–knee–ankle angle (aHKA) were calculated. Intra- and interobserver reliabilities were assessed using ICCs. For interobserver reliability, an experienced senior orthopedic surgeon and an orthopedic resident independently measured the same set of 50 radiographs (100 knees). For intraobserver reliability, the same observers repeated the measurements of these 50 radiographs after a 1-month interval. Interobserver reliability was evaluated using the ICC (2,1) model. All parameters demonstrated high reliability, with ICC values exceeding 0.75 (range, 0.87–0.99) [11].

### 2.3 Clinical scoring system

Subjective knee symptoms were assessed using the Knee Injury and Osteoarthritis Outcome Score (KOOS), a widely used patient-reported outcome measure [12,13]. The KOOS comprises 42 knee-related items, each scored from 0 to 4. The summed scores of the five subscales (pain, symptoms, ADL, sports/recreation, and QOL) were converted to a 0–100 scale, with higher scores indicating a better condition. The reliability and internal consistency are reportedly high [14]. Physical activity was assessed using a lifestyle questionnaire and the 25-question Geriatric Locomotive Function Scale (GLFS-25).

Health-related quality of life was evaluated using the MOS 36-Item Short-Form Health Survey (SF-36). Depressive symptoms were assessed using the Center for Epidemiological Studies Depression Scale. Cognitive function was assessed using the MCI Screen, while sleep habits were assessed using the Japanese version of the Epworth Sleepiness Scale and the Japanese version of the REM Sleep Behavior Disorder Screening Questionnaire (RBDSQ-J).

Social status was assessed based on family structure, educational attainment, and Lubben Social Network Scale-6 (LSNS-6). Urinary function was evaluated using the International Prostate Symptom Score and Overactive Bladder Symptom Score. Bowel habits were assessed using the Bristol Stool Scale. Information on past medical and surgical history, current symptoms, primary care physicians, prescribed medications, and use of health supplements was collected. In addition, educational attainment and occupational status including secondary employment were also documented.

### 2.4 Bone mineral density (BMD) measurements

BMD at one-third distal radius was measured using dual-energy X-ray absorptiometry with the DCS-600EXV (Hitachi Aloka Medical, Tokyo, Japan), based on previous reports [15,16]. Briefly, the region of interest was measured on the non-dominant side unless a fracture was present, in which case, measurements were taken on the dominant side.

### 2.5 Blood examination

Blood and urine samples were collected from all participants after an overnight fast of at least 10 h. Analyses were performed by LSI Medience Corporation (Tokyo, Japan), an ISO-15189-certified laboratory, under strict quality control. Laboratory analyses included a complete blood count, serum biochemistry, amino acid profiling, fatty acid profiling, lymphocyte subset analysis, electrolyte levels, creatinine, metanephrine, and normetanephrine levels. Urinalysis included measurements of electrolytes, albumin, creatinine, cortisol, metanephrine, and normetanephrine levels. In addition, salivary concentration and secretion rate of immunoglobulin A were measured.

### 2.6 Body composition

Chest, waist, and hip circumferences were measured in all participants. Body composition was evaluated using a multifrequency bioelectrical impedance analyzer (InBody 770, InBody Co., Ltd., Seoul, Korea). The InBody 770 utilizes a multifrequency segmental measurement technique with an 8-point tactile electrode system. Measurements were conducted at multiple frequencies (1, 5, 50, 250, 500, and 1000 kHz) for each body segment (arms, trunk, and legs). This analyzer demonstrated a strong correlation with the gold standard method of dual-energy X-ray absorptiometry [17].

Blood pressure was measured in all four limbs, and vascular function was assessed using the ankle-brachial index and cardio–ankle vascular index. Physical examination of the knee included assessment of tenderness at the medial and lateral joint lines, presence of joint effusion, range of motion of the knee joint, the Thessaly test [18], and evaluation of pain distribution around the knee using a pain-drawing system [19]. Physical function was assessed using handgrip strength, the 10-m maximum walking speed test, and sit-to-stand test.

### 2.7 Lifestyle habits

Dietary habits were evaluated using a food habits and preference questionnaire and the Food Frequency Questionnaire. The Food Frequency Questionnaire evaluated portion size and consumption frequency for each food item. For portion size, a reference indicator was provided for each question (e.g., three slices of thinly sliced pork for stir-fried pork). Participants respond by selecting one for a smaller amount (less than half of the reference), two for an equivalent amount, and three for a larger amount (1.5 times or more). Consumption frequency was evaluated on a nine-point scale ranging from less than once per month to seven or more times per day. Meat items were evaluated separately according to the preparation method. In addition, participants were surveyed regarding their attitudes toward dietary habits, as well as their alcohol consumption, and smoking behaviors.

For physical activity, the type, frequency, and duration of exercise performed during winter and non-winter seasons were recorded.

Details regarding the units and coding schemes for each variable are provided in a separate supplementary file.

### 2.8 Statistical analysis

Demographic data were analyzed using SPSS software (version 30.0; SPSS Inc., Chicago, IL, USA). The chi-square test was used to compare the proportion of women, and the Mann-Whitney *U* test was used to compare age, body mass index (BMI), and BMD between the non-KOA and KOA groups. Statistical *p*-value below 0.05 was considered statistically significant.

To facilitate the interpretation of the results and reduce the computational burden of the BN analysis, background factors associated with the presence or absence of KOA were narrowed down in overall study population. In addition, as a subgroup analysis, the same feature selection procedure was performed in women. First, a machine-learning-based approach using DataRobot (DataRobot Inc., Boston, MA, USA) was adopted to select features associated with KOA. DataRobot has increasingly been applied in the medical field to develop predictive models and to identify featured factors [20,21]. We constructed binary classification models for the KOA. Given the relatively small sample size, no holdout set was used in order to preserve a sufficient number of participants for model development. In addition, external validation was difficult because the present study incorporated a wide range of variables. To minimize the potential impact of overfitting, tenfold cross-validation was performed during model development. For the feature selection, the Boruta algorithm [22] was applied to improve the explanatory capability. Ten random noise variables were introduced into the dataset, and tenfold cross-validation was performed with ten different random seeds. Ensemble models were generated from each partitioned dataset, and the permutation importance, a commonly used criterion for estimating variable importance in prediction, was calculated. Variables with a permutation importance lower than the median value of random noise variables were excluded. This process was repeated five times using random seeds. Ensemble models were constructed using only the remaining features, and their performance (AUC) and permutation importance were evaluated. The reported AUC values were obtained after cross-validation. The relationship between each factor and the model predictions was evaluated using partial dependence plots (PDPs), which are presented in the Supplementary Material. For comparison with the developed models, a reference logistic regression model including age, sex, and BMI as independent variables was constructed, and its AUC was calculated.

BN analysis was conducted to explore the relationships between the identified factors and KOA. A BN is a probabilistic graphical model that represents conditional dependencies among multiple variables using a directed acyclic graph. Directed edges (arcs) estimated in the network can be interpreted as potential causal relationships. BN has been effectively applied to model various systems, including gene regulatory networks [23,24], transcriptome networks [25], protein signaling pathways [26], and medical diagnostic processes [27]. Furthermore, it has recently been used to model the relationships between many variables in health examination data [28] [29].

We used the B-spline nonparametric regression version of BN [23], which flexibly models the nonlinear continuous relationship among variables. We employed the greedy hill-climbing algorithm with bootstrapping [30], as this method efficiently handles many variables. To obtain a stable network structure, we performed 1000 bootstrap resamplings and estimated the network structure for each resampled dataset. The resulting networks were integrated by retaining only edges that appeared with a frequency above a predefined threshold. Given the exploratory nature of this study, the threshold was set at 5% to retain as many potentially relevant network structures surrounding KOA as possible. The analysis was performed using the INGOR software [23], [31]. After estimating the BN structure, both the entire network and the relationships directly connected to KOA were visualized using Cytoscape [32]. The Markov blanket of the KOA was extracted for factors directly associated with the KOA (Fig. 1).

Finally, logistic regression analysis was performed with the presence or absence of KOA as the dependent variable and sex, age, BMI, and dietary habit–related variables identified in the overall study population as independent variables. Odds ratios, 95% confidence intervals, and corresponding *p*-values were estimated.

## 3 Results

### 3.1 Demographics

Among the 703 participants, 449 (63.9%) were classified as non-KOA and 254 (36.1%) as KOA. The mean age was significantly higher in the KOA group than in the non-KOA group *(p* < 0.001). The proportion of women was also significantly higher in the KOA group than in the non-KOA group *(p* < 0.001). BMD was significantly lower in the KOA group than in the non-KOA group (*p* < 0.001). All KOOS subscale scores were significantly lower in the KOA group than in the non-KOA group (*p* < 0.001). No significant difference in BMI was observed between the two groups (Table 1).

**Table 1:** Demographic and pain-related variables are presented as mean ± standard deviation for the non-KOA and KOA groups. Continuous variables were compared using the Mann–Whitney U test, and the proportion of women was compared using the chi-squared test.

	Non-KOA	KOA	P-value

Numbers	449	254	–
Age	49.0 ± 14.2	60.0 ± 13.8	<0.001
Female (%)	227 (50.6)	186 (73.2)	<0.001
BMI	23.1 ± 3.5	23.1 ± 3.2	0.903
BMD (%)	98.6 ± 11.4	91.8 ± 15.2	<0.001
KOOS symptom	94.4 ± 8.4	85.2 ± 16.8	<0.001
KOOS pain	96.4 ± 9.0	87.1 ± 16.3	<0.001
KOOS QOL	91.0 ± 14.9	74.5 ± 23.8	<0.001
KOOS ADL	98.4 ± 6.1	93.1 ± 11.7	<0.001
KOOS sports	95.4 ± 12.8	81.1 ± 25.1	<0.001

**Fig. 2:**
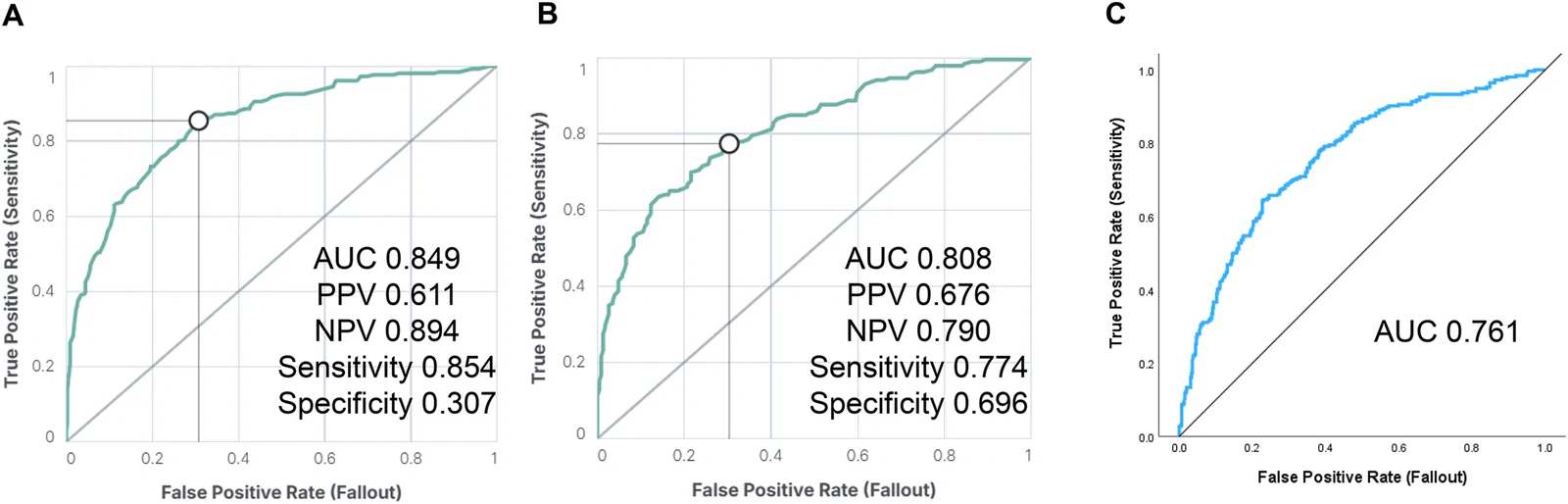
A. Receiver operating characteristic (ROC) curve of the model with the highest AUC in overall population. B. ROC curve of the model with the highest AUC in women. C. ROC curve of the reference logistic regression model including age, sex, and BMI in the overall population. The AUC was 0.761.

**Fig. 3:**
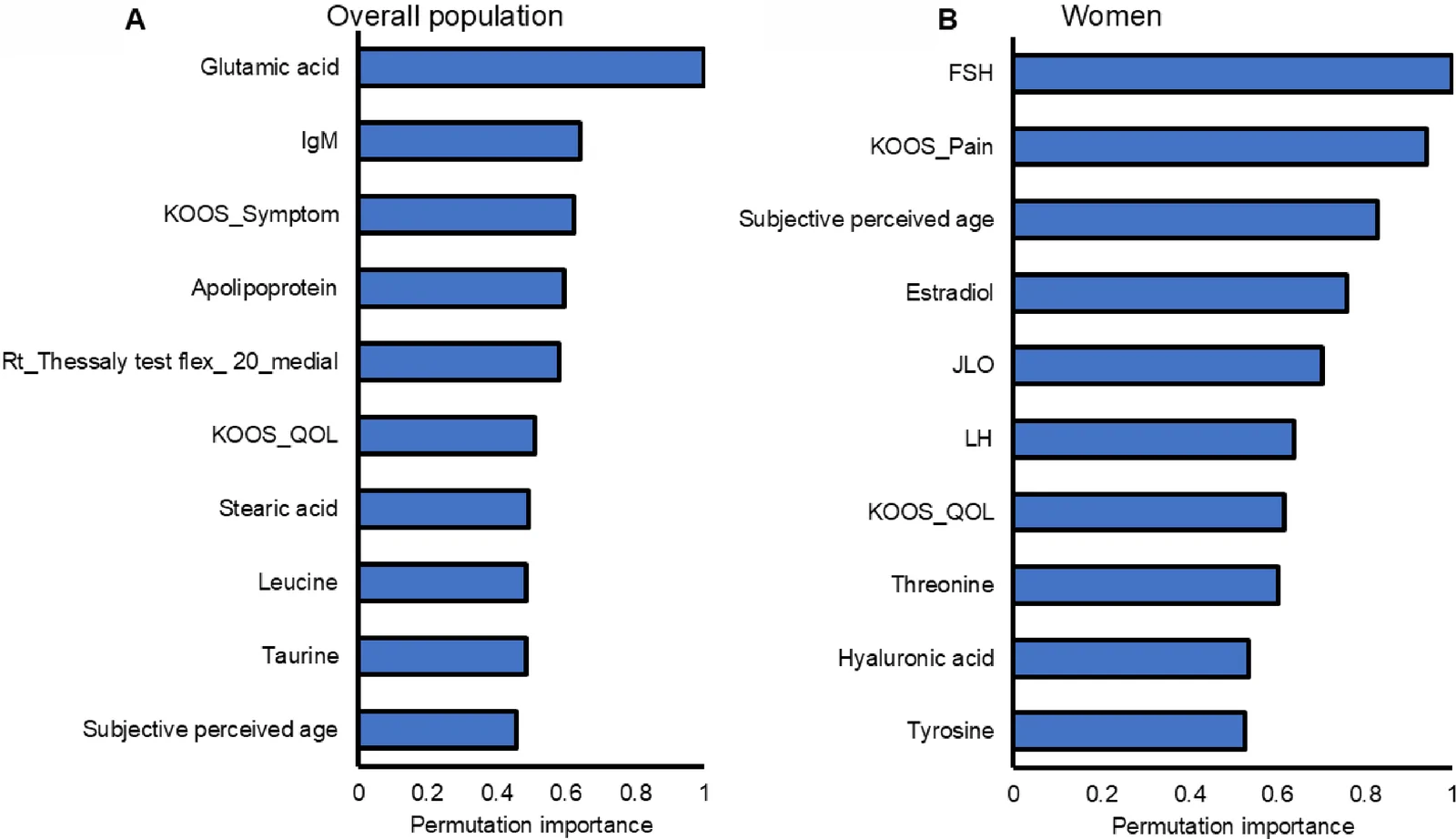
A. Top 10 variables ranked by permutation importance in the model for the overall population. Variables related to amino acids, knee symptoms, fatty acid and age were among the top-ranked features. B. Top 10 variables ranked by permutation importance in the model for women. Variables related to hypothalamic–pituitary–gonadal axis hormones, knee symptoms, age, lower limb alignment, and amino acids were among the top-ranked features.

From the initial 993 background factors, 50 variables in the overall study population and 54 variables in women were retained following the selection. Ensemble classification models were constructed using these variables. In the overall study population, the best-performing model was an ensemble model combining a Nystroem Kernel Support Vector Machine classifier, an Extreme Gradient Boosted Trees classifier, and a Random Forest classifier. In women, the best-performing model was a Light Gradient Boosted Trees classifier with standardization and an Elastic-Net classifier. The corresponding AUC were 0.849 in the overall study population (Fig. 2A) and 0.808 in women (Fig. 2B). The reference logistic regression model comprising age, sex, and BMI had an AUC of 0.761 (Fig. 2C). The top 10 variables ranked by permutation importance were factors related to fatty acid, amino acid levels, and knee symptoms in the overall study population (Fig. 3A). In addition, knee symptoms, amino acid levels and hypothalamic-pituitary-gonadal axis hormone levels were highest in women (Fig. 3B). The relationships between each factor and the model predictions and schematic overviews of the model-development process are presented in the Supplementary Material 1, 2, 3 and 4.

**Fig. 4:**
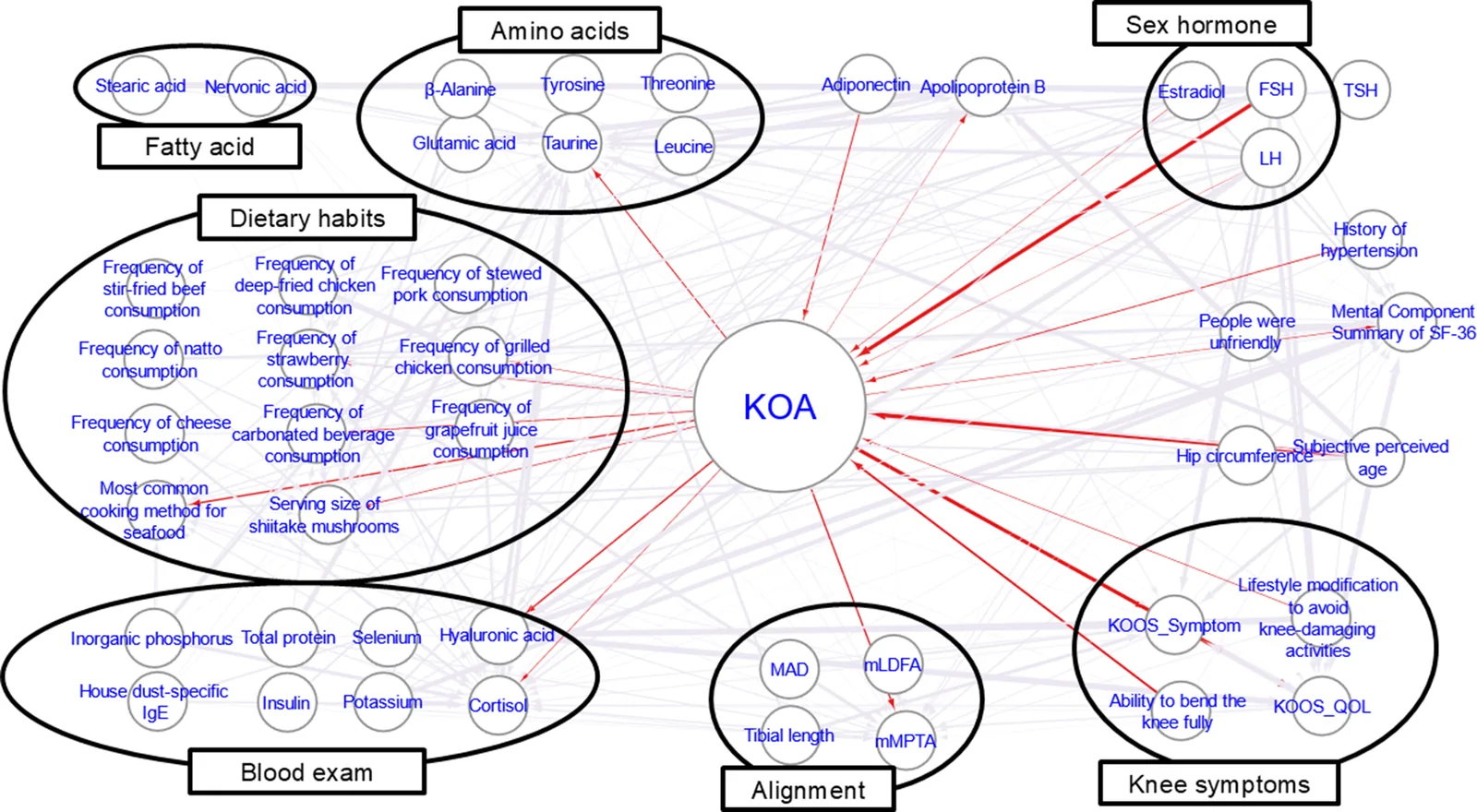
Items located in the Markov blanket of KOA within the BN in overall population. Red arrows indicate variables directly associated with KOA. Variables upstream of an arrow represent parent nodes, whereas those downstream represent child nodes.

**Fig. 5:**
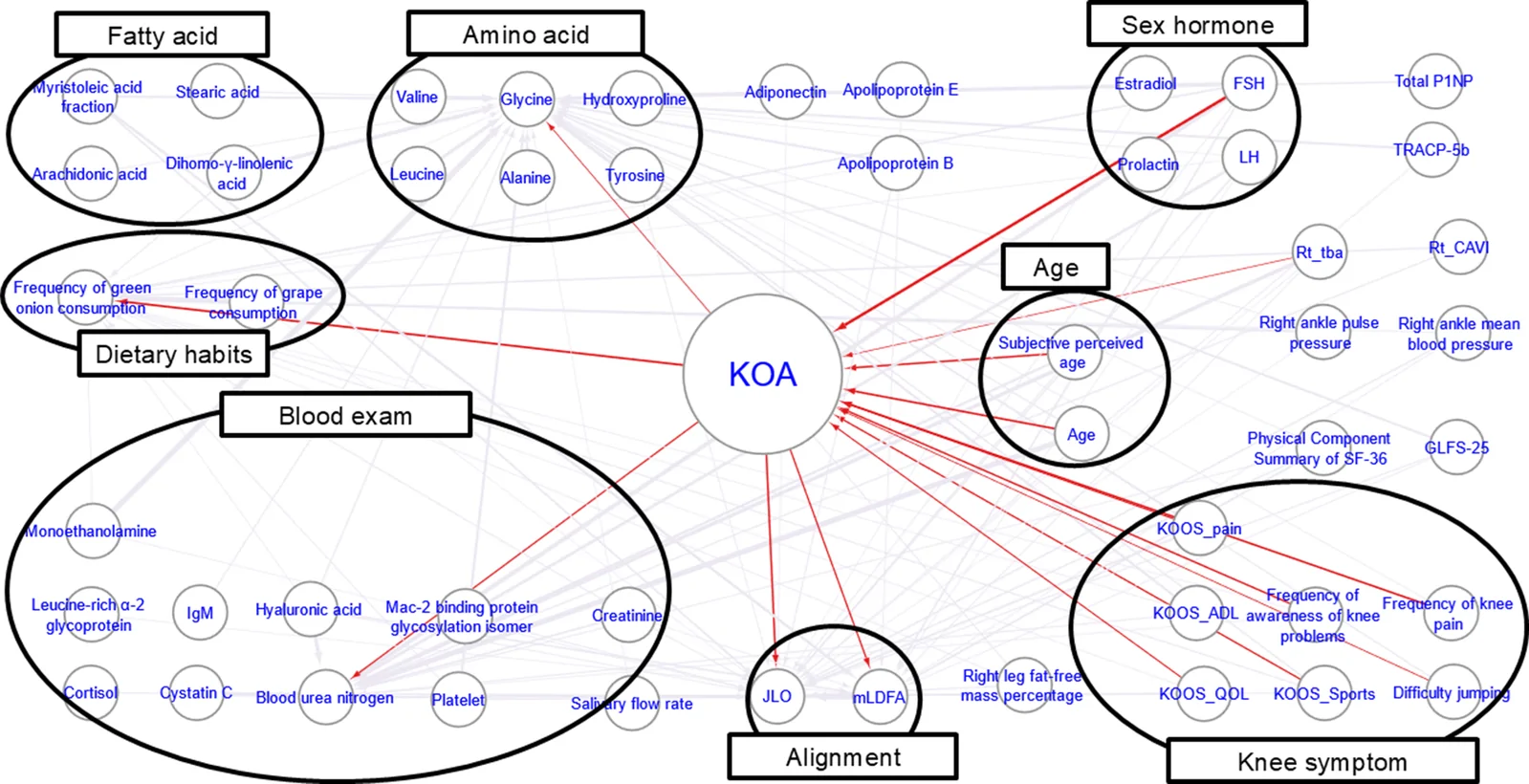
Items located in the Markov blanket of KOA within the BN in women. Red arrows indicate variables directly associated with KOA. Variables upstream of an arrow represent parent nodes, whereas those downstream represent child nodes.

The nodes and edges within the Markov blanket of KOA in the overall study population and in women are shown in Fig. 4, Fig. 5, respectively. Edge thickness is proportional to the bootstrap probability, and red edges indicate direct connections to KOA. Supplementary material 5 and 6 show only the edges directly connected to KOA in the overall study population and in women, respectively. Orange nodes are located upstream of KOA in the estimated network, whereas green nodes are located downstream of KOA. These directions represent the structure of the estimated BN and do not imply temporal or causal relationships. In both the overall population and women, variables related to knee symptoms, lower limb alignment, sex hormones, amino acids, fatty acids, and dietary habits were identified as being associated with KOA. Notably, in the overall population, a relatively large number of dietary habit–related variables were identified.

In the logistic regression analysis including dietary habit–related variables retained in the overall analysis, the frequency of carbonated beverage consumption and the frequency of deep-fried chicken consumption were significantly associated with KOA (Table 2).

**Table 2:** Related factors for KOA.

	Univariable logistic regression analysis			Multivariable logistic regression analysis		
	β	OR (95% CI)	P-value	β	OR (95% CI)	P-value

Sex	2.675	1.916–3.735	<0.001	3.078	2.078–4.561	<0.001
Age	1.056	1.043–1.068	<0.001	1.058	1.044–1.073	<0.001
BMI	1.000	1.000–1.000	0.686	–	–	–
Frequency of stewed pork consumption	0.990	0.795–1.232	0.927	–	–	–
Frequency of carbonated beverage consumption	0.716	0.620–0.826	<0.001	0.820	0.703–0.956	0.011
Serving size of dried whitebait	1.243	1.053–1.468	<0.001	1.179	0.968–1.437	0.102
Serving size of shiitake mushrooms	1.411	1.181–1.686	<0.001	1.224	1.157–1.768	0.052
Frequency of stir-fried beef consumption	0.913	0.777–1.072	0.268	–	–	–
Frequency of deep-fried chicken consumption	1.242	1.034–1.493	0.021	1.430	1.157–1.768	<0.001
Frequency of cheese consumption	0.986	0.889–1.093	0.783	–	–	–
Frequency of grilled chicken consumption	1.197	0.994–1.441	0.057	–	–	–
Frequency of strawberry consumption	1.360	1.212–1.527	<0.001	1.110	0.976–1.262	0.113
Frequency of grapefruit juice consumption	0.714	0.353–1.446	0.350	–	–	–
Frequency of natto consumption	1.204	1.094–1.325	＜0.001	1.070	0.957–1.196	0.237

Statistical analysis – linear regression analysis. Dependent variable: KOA. Independent variables – sex, age, BMI, BMD, dietary habit related factors identified in the analysis among overall population.

## 4 Discussion

The most important finding of this study was that the AI analysis using comprehensive health data from a large-sample cohort successfully developed a binary classification model to discriminate KOA from non-KOA with extremely high accuracy, achieving AUC values of 0.849 for overall population and 0.808 for women. Among the variables with high permutation importance, those related to amino acids, knee symptoms, fatty acids, and age were identified in the overall population, whereas variables related to hypothalamic–pituitary–gonadal axis hormones, knee symptoms, age, lower limb alignment, amino acids, and hyaluronic acid were identified in women. In the BN analysis, variables related to knee symptoms, sex hormones, amino acids, fatty acids, and dietary habits were associated with KOA in both the overall population and women. Notably, a relatively large number of dietary habit–related variables were identified in the overall population.

### 4.1 Dietary habits

In the present study, various dietary habit–related variables were identified in the BN analysis, particularly in the overall population, with many of these variables related to meat consumption. Notably, the frequency of deep-fried chicken consumption showed a positive relationship with the model prediction of KOA and was also significantly positively associated with KOA in the logistic regression analysis. At this moment, there are few reports investigating the relationship between dietary habits and KOA. Nevertheless, several studies have shown that the intake of milk, muesli, dried fruit, cheese, and oily fish is negatively correlated with the incidence of KOA, whereas coffee consumption is positively correlated [[33], [34], [35]]. Lu et al. examined the association between dietary fat intake and medial joint space width (JSW) on radiographs, reporting that higher intake of total fat and saturated fatty acids was significantly associated with greater JSW narrowing, whereas higher intake and proportions of monounsaturated and polyunsaturated fatty acids were associated with reduced JSW narrowing [36]. Furthermore, Deng et al. demonstrated that dietary polyunsaturated fatty acid intake was effective in alleviating pain and improving joint function in patients with KOA [37]. In the present study, many of the dietary habit–related variables associated with KOA involved foods with a high fat content. As discussed below, fatty acids were also identified as potential factors associated with KOA. Taken together, these findings suggest that fatty acids may be involved in the association between dietary habits, particularly the consumption of high-fat foods, and KOA. However, the temporal and causal relationships underlying these associations need to be further investigated in longitudinal studies.

### 4.2 Amino acid fractions

In the present study, several amino acid fractions were associated with KOA. In the DataRobot analysis, glutamic acid, leucine, and taurine ranked among the variables with high permutation importance in the overall population, whereas threonine and tyrosine ranked highly in women. In the BN analysis, taurine in the overall population and glycine in women were directly connected to KOA. Leucine and threonine are essential amino acids, whereas glutamic acid and tyrosine are non-essential amino acids. Taurine is a sulfur-containing amino acid. Several *in vitro* studies have suggested that leucine and taurine may exert protective effects on chondrocytes [38,39]. In contrast, *in vitro* evidence suggests that glutamate may be involved in inflammatory processes associated with cartilage degeneration through N-methyl-d-aspartate (NMDA) receptors in the knee joint [40]. With regard to tyrosine, nitrotyrosine, a product of tyrosine nitration, has been reported to be elevated in the synovial fluid and plasma of patients with OA [41]. Evidence directly linking threonine to structural KOA remains limited. Although the present findings suggest associations between these amino acids and KOA, their biological significance and causal relationships remain unclear and warrant further investigation.

### 4.3 Fatty acid

In the present study, stearic acid showed high permutation importance in the DataRobot analysis of the overall population. In the BN analysis, several fatty acids, including arachidonic acid, were identified within the Markov blanket of KOA, suggesting conditional dependencies with KOA. Stearic acid is a saturated fatty acid, and several studies have suggested that it may contribute to the production of pro-inflammatory cytokines and promote apoptosis in chondrocytes [42]. Arachidonic acid is an omega-6 polyunsaturated fatty acid. In a human observational study, Baker et al. reported that serum n-3 fatty acid concentrations were negatively associated with patellofemoral cartilage loss, whereas arachidonic acid concentrations were positively associated with synovitis [43]. In contrast, Felson et al. found no significant association between serum n-3 or n-6 fatty acid concentrations and the incidence of KOA [44]. In the present study, partial dependence plots (PDPs) showed that increasing concentrations of both stearic acid and arachidonic acid were associated with a higher predicted probability of KOA. These findings suggest potential associations of stearic acid and arachidonic acid with KOA; however, further studies are needed to clarify these relationships.

### 4.4 Limitations

This study has several limitations. First, because of its cross-sectional design, it is not possible to determine whether the factors identified as being associated with KOA are causes or consequences of the disease. Second, the study participants were community-dwelling individuals with relatively high health awareness, and the study population was characterized by relatively low proportions of participants with severe KOA and obesity and a relatively high proportion of women, which may have introduced selection bias. Third, the sample size was relatively small in relation to the large number of variables included in the analysis. Fourth, no holdout set was used in order to preserve a sufficient sample size for model development. Fifth, external validation was not performed. Although 10-fold cross-validation was employed to mitigate the risk of overfitting, the possibility of overfitting cannot be completely excluded. Sixth, the network structures derived from the BN analysis represent conditional dependence structures among the variables; therefore, the direction of an edge should not be interpreted as indicating temporal order or a causal relationship. Seventh, because dietary habits were assessed using a self-administered questionnaire, the possibility of recall bias cannot be excluded.

## 5 Conclusion

In conclusion, AI analysis using comprehensive health data from a community-based cohort enabled the development of a binary classification model that discriminated between KOA and non-KOA with high accuracy. Amino acids, fatty acids, dietary habits, and knee symptoms were among the variables associated with KOA in the prediction models and BN analysis. These findings should be considered exploratory, and further studies are needed to validate these associations.

## Ethics approval and informed consent

The study was performed in accordance with the 1964 Declaration of Helsinki and its later amendments or comparable ethical standards. Approval was obtained from the ethics committee of Hirosaki University Graduate School of Medicine (May 29, 2024; approval number 2024–016). Written informed consent was obtained from all participants.

## Author contributions

All authors made a significant contribution to the work reported, whether that is in the conception, study design, execution, acquisition of data, analysis, and interpretation, or in all these areas; took part in drafting, revising, or critically reviewing the article; gave final approval of the version to be published; agreed on the journal to which the article has been submitted; and agreed to be accountable for all aspects of the work.

## Consent for publication

All authors have read and approved the final version of this manuscript for publication.

## Data availability

Data cannot be shared publicly because of ethical concerns. Data are available from the Hirosaki University COI Institutional Data Access/Ethics Committee (contact via e-mail: coi@hirosaki-u.ac.jp) for researchers who meet the criteria for data access. Researchers need to be approved by the research ethics review board of the organization of their affiliations.

## Declaration of generative AI and AI-assisted technologies in the manuscript preparation process

During the preparation of this work the author(s) used ChatGPT in order to use for English language editing. After using this tool/service, the author(s) reviewed and edited the content as needed and take(s) full responsibility for the content of the published article.

## Role of the Funding source

This work was supported by Japan Science and Technology Agency (JST) Grant Numbers JPMJCE1302, JPMJCA2201, and JPMJPF2210.

## Conflict of interest

The authors declare no competing interests.
