# Supplementary material for: Multifactorial contributors to knee osteoarthritis identified by Machine Learning and Bayesian network analysis: A cross-sectional study of the Iwaki Health Promotion Project
Source: Osteoarthr Cartil Open. 2026 Sep 7;8(4):100874. doi: 10.1016/j.ocarto.2026.100874 (PMC13601055; doi:10.1016/j.ocarto.2026.100874)
Supplement: Supplementary file 1 [file mmc3.docx]

The units and coding schemes for each variable are presented below.

| **Variable** | **Unit** | **Coding** | **Transfromation** |
| --- | --- | --- | --- |
| Sex | - | Continuous | None |
| Age | years | Male = 0, Female = 1 | None |
| Height | cm | Continuous | None |
| Lower limb alignment | degree | Continuous | None |
| Weight | kg | Continuous | None |
| Body circumference mesurements | cm | Continuous | None |
| Handedness | - | Right ＝1, Left = 2 | None |
| 2 Step test | cm | Continuous | None |
| Grip strength | kg | Continuous | None |
| Optimal walking speed | m/s | Continuous | None |
| Maximum walking speed | m/s | Continuous | None |
| Leg use during the sit-to-stand test | - | Both legs = 1, Single leg = 2 | None |
| Seat height during the sit-to-stand test | - | 40cm = 1; 30cm = 2; 20cm = 3; 10cm = 4 | None |
| Blood pressure–related parameters | mmHg | Continuous | None |
| Medical history–related variables | - | No medical history = 1; Medical history with medication = 2; Medical history without medication = 3 | None |
| Presence of a primary care physician | - | Yes = 1; No = 2 | None |
| Oral Health Impact Profile – related variables | - | Never = 1; Hardly ever = 2; Occasionally = 3; Fairly often = 4; Very often = 5 | None |
| Presence of nasal symptoms | - | Yes = 1; No = 2 | None |
| Presence of hand eczema | - | Yes = 1; No = 2 | None |
| History of stumbling | - | Yes = 1; No = 2 | None |
| History of falls | - | Yes = 1; No = 2 | None |
| Self-reported knee alignment | - | Severe varus = 1; Varus = 2; Straight = 3; Valgus = 4; Severe valgus = 5 | None |
| GLFS-25 items related to pain, discomfort, anxiety and difficulty | - | None = 1; Mild = 2; Moderate = 3; Considerable = 4; Severe = 5 | None |
| GLFS-25 item related to continuous walking distance | - | ≥2–3 km = 1; Approximately 1 km = 2; Approximately 300 m = 3; Approximately 100 m = 4; Approximately 10 m = 5 | None |
| GLFS-25 items related to social participation | - | Not restricted = 1; Slightly restricted = 2; Moderately restricted = 3; Considerably restricted = 4; Completely restricted = 5 | None |
| Use of prescription medications | - | Yes = 1; No = 2 | None |
| Use of over-the-counter medications | - | Yes = 1; No = 2 | None |
| Subjective happiness | - | 7-point scale: 1 = Least happy; 7 = Most happy | None |
| Consumption of foods containing lactic acid bacteria | - | Yes = 1; No = 2 | None |
| Breakfast consumption | - | Yes = 1; No = 0 | None |
| Lunch consumption | - | Yes = 1; No = 0 | None |
| Dinner consumption | - | Yes = 1; No = 0 | None |
| Frequency of snacking | - | None = 1; Less than once per week = 2; 1–2 times per week = 3; ≥3 times per week = 4 | None |
| Dining companion at breakfast | - | Family = 1; Spouse/partner only = 2; Friends/colleagues = 3; Alone = 4; Does not eat the meal = 5 | None |
| Dining companion at lunch | - | Family = 1; Spouse/partner only = 2; Friends/colleagues = 3; Alone = 4; Does not eat the meal = 5 | None |
| Dining companion at dinner | - | Family = 1; Spouse/partner only = 2; Friends/colleagues = 3; Alone = 4; Does not eat the meal = 5 | None |
| Location of breakfast | - | Home = 1; Restaurant/eating out = 2; Workplace or other location away from home = 3; Does not eat the meal = 4; Other = 5 | None |
| Location of lunch | - | Home = 1; Restaurant/eating out = 2; Workplace or other location away from home = 3; Does not eat the meal = 4; Other = 5 | None |
| Location of dinner | - | Home = 1; Restaurant/eating out = 2; Workplace or other location away from home = 3; Does not eat the meal = 4; Other = 5 | None |
| Frequency of eating out | - | Daily = 1; 5–6 days per week = 2; 3–4 days per week = 3; 1–2 days per week = 4; Rarely = 5 | None |
| Frequency of consumption of commercially prepared foods | - | Daily = 1; 5–6 days per week = 2; 3–4 days per week = 3; 1–2 days per week = 4; Rarely = 5 | None |
| Intention to improve dietary habits | - | No intention to improve dietary habits = 1; Intends to improve dietary habits in the future (within approximately 6 months) = 2; Intends to improve dietary habits soon (within approximately 1 month) and has begun making some changes = 3; Has been actively improving dietary habits for <6 months = 4; Has been actively improving dietary habits for ≥6 months = 5 | None |
| Variable category: Other dietary habit–related items | - | Applies = 1; Somewhat applies = 2; Does not really apply = 3; Does not apply = 4 | None |
| Brinkman index | cigarette-years | Continuous | None |
| Secondhand smoke exposure | - | Yes = 1; No = 2 | None |
| Alcohol consumption | - | Yes = 1; No = 2 | None |
| Frequency of alcohol consumption | days/week | Continuous | None |
| Habit of light physical activity | - | Daily = 1; 5–6 days per week = 2; 2–4 days per week = 3; ≤1 day per week = 4; None = 5 | None |
| Exercise during winter | - | Yes = 1; No = 2 | None |
| Exercise during non-winter seasons | - | Yes = 1; No = 2 | None |
| Japanese version of the Epworth Sleepiness Scale (JESS) items | - | Almost never = 1; Slight chance = 2; Moderate chance = 3; High chance = 4 | None |
| Method of completing the RBDSQ-J | - | Completed by the participant alone = 1; Completed in consultation with a family member or bed partner = 2 | None |
| Other RBDSQ-J items | - | Yes = 1; No = 2 | None |
| Subjective age | years | Continuous | None |
| Presence of noise exposure | - | Yes = 1; No = 2 | None |
| Highest educational attainment | - | Elementary school = 1; Junior high school = 2; High school = 3; Junior college/vocational school = 4; University or higher = 5; Other = 6 | None |
| Number of household members | persons | Continuous | None |
| Marital status | - | Married = 1; Widowed = 2; Divorced = 3; Never married = 4 | None |
| Lubben Social Network Scale-6 (LSNS-6) items | persons | Continuous | None |
| Number of people available for support or care | persons | Continuous | None |
| Occupation | - | Professional/technical worker = 1; Managerial worker = 2; Clerical worker = 3; Sales worker = 4; Service worker = 5; Security worker = 6; Agricultural worker = 7; Forestry worker = 8; Fishery worker = 9; Transportation/communication worker = 10; Production/process and labor worker = 11; Homemaker = 12; Unemployed = 13; Student = 14; Unclassifiable = 15 | None |
| Working hours | hours | Continuous | None |
| Employment status | - | Self-employed = 1; Full-time employee = 2; Part-time/casual employee = 3; Contract/temporary agency employee = 4; Not applicable = 5 | None |
| Secondary employment | - | Yes = 1; No = 2 | None |
| SF-36 item on general health status | - | Excellent = 1; Very good = 2; Good = 3; Fair = 4; Poor = 5 | None |
| SF-36 item comparing current health with one year ago | - | Much better than one year ago = 1; Somewhat better than one year ago = 2; About the same as one year ago = 3; Somewhat worse than one year ago = 4; Much worse than one year ago = 5 | None |
| SF-36 items related to physical functioning | - | Limited a lot = 1; Limited a little = 2; Not limited at all = 3 | None |
| SF-36 item comparing current health with one year ago | - | Much better than one year ago = 1; Somewhat better than one year ago = 2; About the same as one year ago = 3; Somewhat worse than one year ago = 4; Much worse than one year ago = 5 | None |
| SF-36 items related to difficulty in physical activities | - | Very difficult = 1; Somewhat difficult = 2; Not difficult at all = 3 | None |
| SF-36 items related to role limitations due to physical health | - | All of the time = 1; Most of the time = 2; Some of the time = 3; A little of the time = 4; None of the time = 5 | None |
| SF-36 item related to social functioning | - | Not at all = 1; Slightly = 2; Moderately = 3; Quite a bit = 4; Extremely = 5 | None |
| SF-36 item related to frequency of bodily pain | - | None = 1; Very mild = 2; Mild = 3; Moderate = 4; Severe = 5; Very severe = 6 | None |
| SF-36 item related to interference with work due to bodily pain | - | Not at all = 1; Slightly = 2; Moderately = 3; Quite a bit = 4; Extremely = 5 | None |
| SF-36 items related to feelings and emotional well-being | - | All of the time = 1; Most of the time = 2; Some of the time = 3; A little of the time = 4; None of the time = 5 | None |
| SF-36 items related to perceived health | - | Definitely true = 1; Mostly true = 2; Not sure = 3; Mostly false = 4; Definitely false = 5 | None |
| Weight loss of 2–3 kg within the past 6 months | - | Yes = 1; No = 2 | None |
| Unexplained fatigue | - | Yes = 1; No = 2 | None |
| Subjective memory complaints | - | Yes = 1; No = 2 | None |
| Subjective decline in walking speed | - | Yes = 1; No = 2 | None |
| Frequency of visits to hot spring facilities | - | ≥3 times per week = 1; 1–2 times per week = 2; 1–2 times per month = 3; Rarely = 4 | None |
| Center for Epidemiologic Studies Depression Scale (CES-D) items | - | Rarely or none of the time = 1; Some or a little of the time = 2; Occasionally or a moderate amount of the time = 3; Most or all of the time = 4 | None |
| IPSS items related to urinary symptoms | - | Not at all = 1; Less than 1 time in 5 = 2; Less than half the time = 3; About half the time = 4; More than half the time = 5; Almost always = 6 | None |
| IPSS items related to nocturnal urinations | - | 0 times = 1; 1 time = 2; 2 times = 3; 3 times = 4; 4 times = 5; ≥5 times = 6 | None |
| IPSS quality-of-life item related to urinary symptoms | - | Delighted = 1; Pleased = 2; Mostly satisfied = 3; Mixed = 4; Mostly dissatisfied = 5; Unhappy = 6; Terrible = 7 | None |
| Number of daytime urinations | - | ≤7 times = 1; 8–14 times = 2; ≥15 times = 3 | None |
| Frequency of urinary urgency | - | None = 1; Less than once per week = 2; ≥1 time per week = 3; Approximately once per day = 4; 2–4 times per day = 5; ≥5 times per day = 6 | None |
| Frequency of urgency urinary incontinence | - | None = 1; Less than once per week = 2; ≥1 time per week = 3; Approximately once per day = 4; 2–4 times per day = 5; ≥5 times per day = 6 | None |
| Usual stool consistency (Bristol Stool Form Scale) | - | Separate hard lumps, like nuts (difficult to pass) = 1; Sausage-shaped but lumpy = 2; Like a sausage but with cracks on the surface = 3; Like a sausage or snake, smooth and soft = 4; Soft blobs with clear-cut edges (passed easily) = 5; Fluffy pieces with ragged edges, a mushy stool = 6; Watery, no solid pieces, entirely liquid = 7 | None |
| Items related to abdominal pain and bowel movements | - | Yes = 1; No = 2 | None |
| Body composition parameters related to muscle and fat mass | Kg | Continuous | None |
| Body composition parameters related to muscle, fat and water percentages | % | Continuous | None |
| Body composition parameters related to body water | L | Continuous | None |
| BMI | Kg/m/m | Continuous | None |
| Waist Hip Ratio | - | Continuous | None |
| Visceral Fat Area | cm² | Continuous | None |
| Obesity degree | - | Continuous | None |
| Bone_Mineral_Content | kg | Continuous | None |
| Bioelectrical impedance parameters | Ω | Continuous | None |
| Measured Circumference of Righ Thigh | cm | Continuous | None |
| Skeltal mass index | Kg/m/m | Continuous | None |
| Right ankle mean arterial pressure | mmHg | Continuous | None |
| Right ankle pulse pressure | mmHg | Continuous | None |
| Rt-ABI | - | Continuous | None |
| Pulse | Bpm | Continuous | None |
| Pulse wave transit time–related parameters | ms | Continuous | None |
| Pulse wave propagation distance–related parameters | cm | Continuous | None |
| Rt-CAVI | - | Continuous | None |
| Vasucular age | years | Continuous | None |
| Change in dietary habits | - | No change = 1; Changed within the past year = 2; Changed 1–2 years ago = 3; Changed 3–5 years ago = 4 | None |
| Frequency of alcohol consumption | - | <1 day per month = 1; 1–3 days per month = 2; 1–2 days per week = 3; 3–4 days per week = 4; 5–6 days per week = 5; Daily = 6 | None |
| mount of alcohol consumed by beverage type | - | None = 1; <0.5 go = 2; 1 go = 3; 2 go = 4; 3 go = 5; 4 go = 6; 5–6 go = 7; ≥7 go = 8  1 go = approximately 180 mL | None |
| Size of rice bowl usually used | - | Small rice bowl = 1; Standard rice bowl = 2; Large bowl (donburi) = 3 | None |
| Number of bowls of rice consumed | - | <1 bowl = 1; 1 bowl = 2; 2 bowls = 3; 3 bowls = 4; 4 bowls = 5; 5 bowls = 6; 6 bowls = 7; 7–9 bowls = 8; ≥10 bowls = 9 | None |
| Frequency of miso soup consumption | - | Rarely or never = 1; 1–3 days per month = 2; 1–2 days per week = 3; 3–4 days per week = 4; 5–6 days per week = 5; Daily = 6 | None |
| Number of bowls of miso soup consumed | - | <1 bowl = 1; 1 bowl = 2; 2 bowls = 3; 3 bowls = 4; 4 bowls = 5; 5 bowls = 6; 6 bowls = 7; 7–9 bowls = 8; ≥10 bowls = 9 | None |
| Saltiness of miso soup | - | Light = 1; Moderate = 2; Strong = 3 | None |
| Frequency of consumption of each food item | - | <1 time per month = 1; 1–3 times per month = 2; 1–2 times per week = 3; 3–4 times per week = 4; 5–6 times per week = 5; Once per day = 6; 2–3 times per day = 7; 4–6 times per day = 8; ≥7 times per day = 9 | None |
| Amount consumed for each food item | - | Small (≤0.5 times the reference portion) = 1; Same as the reference portion = 2; Large (≥1.5 times the reference portion) = 3 | None |
| Frequency of consumption of each beverage | - | <1 time per week = 1; 1–2 times per week = 2; 3–4 times per week = 3; 5–6 times per week = 4; 1 cup per day = 5; 2–3 cups per day = 6; 4–6 cups per day = 7; 7–9 cups per day = 8; ≥10 cups per day = 9 | None |
| Amount of milk added to tea or coffee | - | None = 1; Half a spoonful = 2; 1 spoonful = 3; 2 spoonfuls = 4; ≥3 spoonfuls = 5 | None |
| Amount of sugar added to tea or coffee | - | None = 1; Half a spoonful = 2; 1 spoonful = 3; 2 spoonfuls = 4; ≥3 spoonfuls = 5 | None |
| Frequency of breakfast consumption | - | <1 time per month = 1; 1–3 times per month = 2; 1–2 times per week = 3; 3–4 times per week = 4; 5–6 times per week = 5; Daily = 6 | None |
| Frequency of eating out | - | <1 time per month = 1; 1–3 times per month = 2; 1–2 times per week = 3; 3–4 times per week = 4; 5–6 times per week = 5; Daily = 6 | None |
| Frequency of instant food consumption | - | <1 time per month = 1; 1–3 times per month = 2; 1–2 times per week = 3; 3–4 times per week = 4; 5–6 times per week = 5; Daily = 6 | None |
| Frequency of stir-fried food consumption | - | <1 time per month = 1; 1–3 times per month = 2; 1–2 times per week = 3; 3–4 times per week = 4; 5–6 times per week = 5; Daily = 6 | None |
| Frequency of fried food consumption | - | <1 time per month = 1; 1–3 times per month = 2; 1–2 times per week = 3; 3–4 times per week = 4; 5–6 times per week = 5; Daily = 6 | None |
| Frequency of use of each seasoning | - | <1 time per month = 1; 1–3 times per month = 2; 1–2 times per week = 3; 3–4 times per week = 4; 5–6 times per week = 5; Daily = 6 | None |
| Type of cooking oil used most frequently | - | Blended vegetable oil = 1; Safflower oil = 2; Corn oil = 3; Soybean oil = 4; Rapeseed/canola oil = 5; Olive oil = 6; Other = 7 | None |
| Most frequently used cooking method for each food item | - | Raw = 1; Boiled/Simmered = 2; Grilled = 3; Deep-fried = 4; Stir-fried = 5; Other = 6 | None |
| Degree of meat doneness | - | Rare = 1; Medium-rare = 2; Medium = 3; Medium-well = 4; Well-done = 5 | None |
| Frequency of grilled fish consumption | - | Rarely or never = 1; 1–3 times per month = 2; 1–2 times per week = 3; 3–4 times per week = 4; 5–6 times per week = 5; Daily = 6 | None |
| Amount of charred portions of grilled fish consumed | - | Rarely or never = 1; Approximately one-third = 2; Approximately half = 3; Approximately two-thirds = 4; Almost all = 5 | None |
| White blood cell count | /μL | Continuous | None |
| Red blood cell count | ×10⁴/μL | Continuous | None |
| Percentage of each leukocyte subtype | % | Continuous | None |
| Platelets | ×10⁴/μL | Continuous | None |
| HbA1c | % | Continuous | None |
| Total bilirubin | Mg/dL | Continuous | None |
| AST | U/L | Continuous | None |
| ALT | U/L | Continuous | None |
| γ-GTP | U/L | Continuous | None |
| Total protein | g/dL | Continuous | None |
| Creatinine | mg/dL | Continuous | None |
| Blood urea nitrogen | mg/dL | Continuous | None |
| Uric acid | mg/dL | Continuous | None |
| Total cholesterol | mg/dL | Continuous | None |
| Triglycerides | mg/dL | Continuous | None |
| Sodium | mEq/L | Continuous | None |
| Potassium | mEq/L | Continuous | None |
| Chloride | mEq/L | Continuous | None |
| Calcium | mg/dL | Continuous | None |
| Inorganic phosphorus | mg/dL | Continuous | None |
| Magnesium | mg/dL | Continuous | None |
| Serum iron | µg/dL | Continuous | None |
| Total iron-binding capacity | µg/dL | Continuous | None |
| Serum copper | µg/dL | Continuous | None |
| Blood glucose | mg/dL | Continuous | None |
| HDL cholesterol | mg/dL | Continuous | None |
| Albumin | g/dL | Continuous | None |
| Unsaturated iron-binding capacity | µg/dL | Continuous | None |
| CRP | mg/dL | Continuous | None |
| IgA | mg/dL | Continuous | None |
| IgG | mg/dL | Continuous | None |
| IgM | mg/dL | Continuous | None |
| C3 | mg/dL | Continuous | None |
| C4 | mg/dL | Continuous | None |
| Apolipoprotein A-I | mg/dL | Continuous | None |
| Apolipoprotein B | mg/dL | Continuous | None |
| Apolipoprotein E | mg/dL | Continuous | None |
| DHEA-S | µg/dL | Continuous | None |
| LDL cholesterol | mg/dL | Continuous | None |
| 1,5-Anhydro-D-glucitol | μg/mL | Continuous | None |
| Hyaluronic acid | ng/mL | Continuous | None |
| Glycated albumin | % | Continuous | None |
| Cystatin C | mg/L | Continuous | None |
| Type IV collagen 7S | ng/mL | Continuous | None |
| Leucine-rich α2-glycoprotein | µg/mL | Continuous | None |
| ALP | U/L | Continuous | None |
| Total P1NP | µg/L | Continuous | None |
| PSA | ng/mL | Continuous | None |
| Prolactin | ng/mL | Continuous | None |
| Progesterone | ng/mL | Continuous | None |
| Insulin | μU/mL | Continuous | None |
| Ferritin | ng/mL | Continuous | None |
| TSH | μIU/mL | Continuous | None |
| LH | mIU/mL | Continuous | None |
| FSH | mIU/mL | Continuous | None |
| C-peptide | ng/mL | Continuous | None |
| Estradiol | pg/mL | Continuous | None |
| Mac-2 binding protein glycosylation isomer | C.O.I. | Continuous | None |
| Allergen-specific IgE concentration | UA/mL | Continuous | None |
| Osmolality | mOsm/kg H₂O | Continuous | None |
| Selenium | µg/dL | Continuous | None |
| Zinc | µg/dL | Continuous | None |
| TRACP-5b | mU/dL | Continuous | None |
| Testosterone | ng/dL | Continuous | None |
| Adiponectin | μg/mL | Continuous | None |
| NTx | nmol BCE/L | Continuous | None |
| IGF-1 | ng/mL | Continuous | None |
| Cortisol | μg/dL | Continuous | None |
| ACTH | pg/mL | Continuous | None |
| Aldosterone | pg/mL | Continuous | None |
| Renin | pg/mL | Continuous | None |
| Vasopressin | pg/mL | Continuous | None |
| Leptin | ng/mL | Continuous | None |
| Pancreatic glucagon | pg/mL | Continuous | None |
| Total plasminogen activator inhibitor-1 | ng/mL | Continuous | None |
| Amino acid profile | μmol/L | Continuous | None |
| Fischer ratio | - | Continuous | None |
| Serotonin | ng/mL | Continuous | None |
| IL-1β | pg/mL | Continuous | None |
| IL-6 | pg/mL | Continuous | None |
| 25-Hydroxyvitamin D2 | ng/mL | Continuous | None |
| 25-Hydroxyvitamin D3 | ng/mL | Continuous | None |
| Fatty acid profile | μg/mL | Continuous | None |
| T/T ratio | - | Continuous | None |
| Eicosapentaenoic acid/arachidonic acid ratio | - | Continuous | None |
| Fatty acid composition | % | Continuous | None |
| TNF-α | pg/mL | Continuous | None |
| Lymphocyte | /uL | Continuous | None |
| Urinary creatinine | mg/dL | Continuous | None |
| Urinary N-acetyl-β-D-glucosaminidase | U/L | Continuous | None |
| Urinary C-peptide | μg/day | Continuous | None |
| Urinary β2-microglobulin | μg/L | Continuous | None |
| Urinary sodium | mEq/L | Continuous | None |
| Urinary potassium | mEq/L | Continuous | None |
| Urinary albumin | mg/L | Continuous | None |
| Urinary creatinine-corrected value | mg/gCr | Continuous | None |
| Urinary cortisol | μg/day | Continuous | None |
| Urinary aldosterone | μg/day | Continuous | None |
| Urinary metanephrine | mg/day | Continuous | None |
| Urinary normetanephrine | mg/day | Continuous | None |
| Urine osmolality | mOsm/kg H₂O | Continuous | None |
| Urinary aquaporin | ng/mL | Continuous | None |
| Salivary IgA concentration | μg/mL | Continuous | None |
| Salivary flow rate | mL/min | Continuous | None |
| Salivary IgA secretion rate | μg/min | Continuous | None |
| KOOS items | - | Scored on a 5-point Likert scale from 0 to 4, with higher scores indicating greater severity of symptoms or difficulty. | None |
| KOOS subscale scores | points | 0–100; higher scores indicate better knee-related health status | None |
| Bone mineral density T-score | % | Continuous | None |
| Knee flexion range of motion | degree | Continuous | None |
| Joint line tenderness by knee | - | Yes = 1; No = 0 | None |
| Thessaly test | - | Positive = 1; Negative = 0 | None |
| Knee pain by region on the pain drawing | - | Yes = 1; No = 0 | None |
| SF-36 subscale scores and summary scores | points | Continuous | None |
